# X-ray source arrays for volumetric imaging during radiotherapy treatment

**DOI:** 10.1038/s41598-023-36708-x

**Published:** 2023-06-16

**Authors:** Owen Dillon, Tess Reynolds, Ricky T. O’Brien

**Affiliations:** 1 Faculty of Medicine and Health, Image X Institute, University of Sydney, Sydney, 2015 Australia; 2 School of Health and Biomedical Sciences, Medical Imaging Facility, Royal Melbourne Institute of Technology, Melbourne, 3083 Australia

**Keywords:** Lung cancer, Radiotherapy

## Abstract

This work presents a novel hardware configuration for radiotherapy systems to enable fast 3D X-ray imaging before and during treatment delivery. Standard external beam radiotherapy linear accelerators (linacs) have a single X-ray source and detector located at ± 90° from the treatment beam respectively. The entire system can be rotated around the patient acquiring multiple 2D X-ray images to create a 3D cone-beam Computed Tomography (CBCT) image before treatment delivery to ensure the tumour and surrounding organs align with the treatment plan. Scanning with a single source is slow relative to patient respiration or breath holds and cannot be performed during treatment delivery, limiting treatment delivery accuracy in the presence of patient motion and excluding some patients from concentrated treatment plans that would be otherwise expected to have improved outcomes. This simulation study investigated whether recent advances in carbon nanotube (CNT) field emission source arrays, high frame rate (60 Hz) flat panel detectors and compressed sensing reconstruction algorithms could circumvent imaging limitations of current linacs. We investigated a novel hardware configuration incorporating source arrays and high frame rate detectors into an otherwise standard linac. We investigated four potential pre-treatment scan protocols that could be achieved in a 17 s breath hold or 2–10 1 s breath holds. Finally, we demonstrated for the first time volumetric X-ray imaging during treatment delivery by using source arrays, high frame rate detectors and compressed sensing. Image quality was assessed quantitatively over the CBCT geometric field of view as well as across each axis through the tumour centroid. Our results demonstrate that source array imaging enables larger volumes to be imaged with acquisitions as short as 1 s albeit with reduced image quality arising from lower photon flux and shorter imaging arcs.

## Introduction

Standard clinical practice in external beam radiotherapy is based around guiding a radiation beam to a target region e.g. cancerous tissue. Tumor motion due to respiration is typically accounted for in lung cancer radiotherapy by expanding the target region to include the entire region the tumor may move during treatment, increasing the volume of tissue irradiated at the cost of additional healthy tissue and side effects. The treatment plan also needs to minimize dose to organs at risk such as the heart. As radiotherapy treatments are now delivering dose more rapidly, for example more dose per treatment session as in Stereotactic Body Radiotherapy (SBRT)^1–4^, or higher dose per second as in FLASH radiotherapy^5–7^, accurate delivery becomes increasingly important and challenging^4,8–10^.

**Figure 1:**
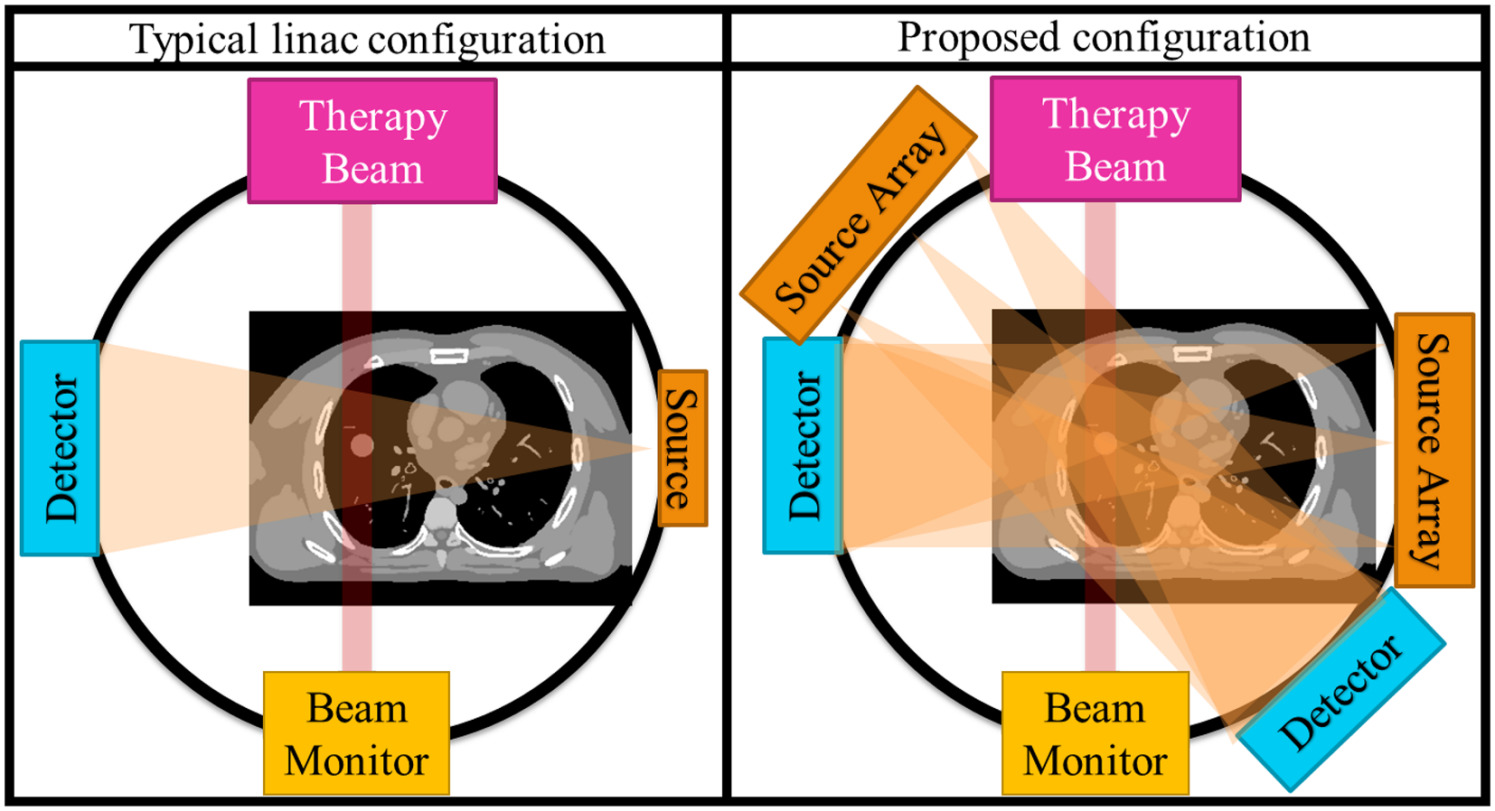
Overview of the configurations considered in this paper. On the left is a conventional linac configuration, with single source offset 90° from the treatment beam. On the right is our proposed configuration, with one source array offset at ${90}^{\circ }$ and another at $-{10}^{\circ }$, each facing a corresponding detector. Note that all labelled hardware in the above figures area attached to the rotating gantry and moves together. Each source array is 60 cm long containing 60 independent sources. The gantry is rotated to a specified angular position then stays still while each source in each array is individually and sequentially activated to acquire each projection image. The gantry is treated as static during intra-treatment acquisition.

A standard radiotherapy linear accelerator (radiotherapy linac) hardware configuration is shown on the left of Fig. 1. The rotating gantry has a Mega Voltage (MV) X-ray source generating the treatment beam, shaped by the Multi Leaf Collimator (MLC) facing an Electronic Portal Imaging Device (EPID). The linear accelerator sends a typically 4–25 MeV electron beam to a tungsten target to produce the 4–25 MeV X-ray treatment beam. These high energy X-rays have good penetration in human tissue, allowing deep tumours to be treated. Typical modern machines have a kilo Voltage (kV) X-ray source for imaging typically located at 90 degrees from the MV source, facing a flat panel detector. The kV source is typically a X-ray tube producing 50–200 keV X-rays for imaging, as X-rays in this spectrum have good contrast for human tissue.

Conventional pre-treatment imaging on a standard linac involves rotating the gantry while acquiring kV projections which are used to reconstruct a Cone Beam Computed Tomography (CBCT) volumetric image. There is a wide range of intra-treatment imaging technologies that may or may not be used depending on the perceived need of intra treatment imaging when treating particular organs for concerns of motion or precision, as well as the presence of such technologies within a given clinic. Intra treatment imaging may include any combination of in room video cameras^11,12^, IR markers on the patient^13^, surface tracking of the patient^11,12,14^, low dose X-ray video using the kV imager (fluoroscopy) or additional external kV imaging systems ^14,15^, or MV imaging using the treatment beam and Electronic Portal Imaging Device^16,17^. External imaging methods can only approximate the motion of internal anatomy^18–23^ Fluoroscopy with the kV imager compresses the anatomy to a 2D plane but at a right angle to the treatment beam. Using the EPID for MV imaging is in the treatment plane but with low contrast and a narrow field of view^16,17^. The need for internal 2D imaging in the treatment plane or 3D imaging during treatment motivates the use of MR-linacs, dedicated integrated MRI scanners inside purpose built radiotherapy linacs, but these systems are relatively expensive and access is limited^7,24–29^.

We propose a novel combination of source arrays and high frame rate detectors as a modification to conventional linacs that addresses limitations on current clinical imaging. Source arrays making use of carbon nanotubes for field emission of X-rays have recently become available and demonstrate sufficient photon energy and flux as well as fast on/off times for X-ray acquisition over very short time frames^30^. These source arrays can be paired with commercially available high frame rate (60–120 Hz) detectors to acquire X-ray projections from several locations in a short time without gantry motion. Such a pairing has been used to create CT systems with no moving parts^30–32^ and is an example of inverse geometry CT^33^.

This work is the first to consider recently available source arrays and high frame rate detectors along with compressed sensing reconstruction as a method of addressing limitations of delivering radiotherapy on current clinical systems, namely the speed of volumetric imaging relative to patient respiration and breath holds. Earlier studies of source arrays for radiotherapy were limited by the relatively small, low flux source arrays, low frame rate detectors, and lower computational resources available at the time^34,35^. These limitations tended to limit applications of source arrays to tomosynthesis rather than full volumetric imaging^35–38^. By using novel hardware to perform volumetric imaging quickly, clinicians can verify 3D anatomy during treatment and safely guide the treatment beam with the high precision required^8^ for emerging high intensity treatments such as SBRT and FLASH^2,3,7,10^.

## Methods

To investigate various potential integrations of source arrays, this work uses a synthetic 4D patient phantom to investigate acquisition using conventional and proposed novel hardware.

### Synthetic patient phantom

To model respiratory motion we used the 4D extended cardiac-torso (XCAT) phantom to generate a ground truth patient with implanted tumor that moves realistically due to respiratory motion^39^. The central coronal slice is shown in Fig. 1 bottom left, with central axial slices shown in the Fig. 1 diagrammatic views. We generated 50 volumes each corresponding to 2% steps through a respiratory cycle with 2 cm diaphragm and 1 cm tumor motion. We simulated the patient in free breathing as having a 5 s respiratory cycle.

### Acquisition simulation

Key scan parameters are recorded in Table 1. In this paper we take a conventional 3D CBCT scan to be 200 projections taken over 35 s in a ${200}^{\circ }$ arc reconstructed with the Feldkamp-Davis-Kress (FDK) algorithm^40^, a variation of Filtered Back Projection (FBP) for cone beam geometry. Other 3D scan protocols are in commercial use for example the Elekta Synergy thoracic CBCT acquires 380 projections over 140 s, Varian TrueBeam up to 900 projections over 60 to 200 s, and the Varian Halcyon hypersight 6 s acquisition, each with modified tube voltage and current to limit overall exposure.

**Table 1:** Condensed description of each scan protocol. Angular separation is the angle between the centre of the source array at each angular position.

	Conventional	120 projections, 4 angular positions	180 projections, 12 angular positions	200 projections, 20 angular positions	1000 projections, 100 angular positions	Intra treatment
Total number of projections	200	120	180	200	1000	120
Angular separation	${1}^{\circ }$	${66}^{\circ }$	${18}^{\circ }$	${10}^{\circ }$	${2}^{\circ }$	${100}^{\circ }$
Projections per angular position	1	30	15	10	10	60
Source separation at each angular position	N\A	2 cm	4 cm	6 cm	6 cm	1 cm
Total photon count	6,000,000	720,000	1,080,000	1,200,000	6,000,000	720,000
photons per projection	30,000	6000	6000	6000	6000	6000
Acquisition time per projection	0.025 s	0.017 s	0.017 s	0.017 s	0.017 s	0.017 s
Acquisition time per angle	0.025 s	0.5 s	0.25 s	0.17 s	0.17 s	1 s
Total scan time	35 s	2 breaths	6 breaths	10 breaths	50 breaths	1 s

Source separation refers to the gap between each source, activated in sequence, in the array at each given angular position. Note that total scan time is not just timer per projection times number of projections as there is time between acquisitions. A conventional scan is 35 s and intra treatment scan 1 s independent of patient breathing while the pre-treatment source array scans are taken to be done in several 1 s breath holds or synchronised with free breathing.

Using a cone beam CT (CBCT) geometry, the forward and back projections, as well as the reconstruction methods, were implemented using the open-source Reconstruction Tool Kit (RTK)^41^. The computational hardware used was a desktop workstation with 64 GB of RAM, 32 3.1 GHz CPU cores and 2 Nvidia GPU cards with combined 3,712 CUDA cores and 16 GB VRAM.

The radiotherapy system was modelled as having a 1000 mm source-isocenter distance, 1,536 mm source-detector distance and full fan geometry as on the Elekta Synergy. We modelled the detector as a 17-inch flat panel with 1mm^2^ pixels in a 430 × 430 pixel array with 60 Hz readout rate.

For the conventional CBCT acquisition attenuation images were converted to intensity images with a 30,000 photon count per projection and corrupted with Poisson noise as in^42,43^, consistent with a typical 0.5 mAs kV image.

The field emission carbon nanotube source arrays are simulated based on^30^. We take each array as being 60 independent and digitally switched sources in a 60 cm array. Note that there is a 1 cm gap between each source, however as in^30^ the focal spot of each source is small relative to a conventional X-ray tube. The proposed configuration is shown in Fig. 1, with one source array at $+{90}^{\circ }$ and the other at $-{10}^{\circ }$ each facing a detector. Source output was taken as 7 mA and we image at 60 Hz for ~ 0.1 mAs per image, so we scale to 6,000 photon count with corresponding Poisson noise.

The array acquisition takes 0.5–1 s at each angular position for each case depending on if all or every second source is used, which we simulate as a Deep Inspiration Breath Hold (DIBH), noting that almost all patients should be able to manage such a short breath hold. Note that the arrays at $+{90}^{\circ }$ and $-{10}^{\circ }$ are operating in parallel.

An advantage in using imagers offset by $100^{ \circ }$ is that a $200^{ \circ }$ arc can be acquired in half the time, in this example we assume the gantry can rotate $200^{ \circ }$ in 35 s for the conventional acquisition so in our proposed configuration the required gantry rotation would take ~ 18 s. If the gantry rotated to each position and stopped while waiting for the patient to perform a 1 s breath hold, the 4 angular position scan would take 19 s. The gantry can reach each angular position of the other scans quickly relative to typical patient 4 s respiratory cycles, so if scans were synchronized as in^43–45^ The 12 and 20 angular position scans would take 24 and 40 s respectively. The 100 angular position scan would take 200 s, comparable to current 4DCBCT protocols, but the time could be reduced further by acquiring from several angular positions per breath hold, with all scans taking 17 s if a 17 s breath hold can be achieved by the patient. Reducing the scan time potentially increases the patient cohort for DIBH pre-treatment imaging due to the shorter scan time but the consistency of multiple breath holds is also an important factor. Note that acquisition over multiple breath holds or synchronized to free breathing can provide clinically relevant information such as breath hold repeatability which factors into patient selection to perform breath hold procedures clinically.

There are infinitely many possible acquisitions to simulate, however for pre-treatment with gantry rotation we restricted our simulations to 120 projections from 4 angular positions, 180 from 12 angular positions, 200 from 20 angular positions, and 1000 from 100 angular positions. Note that e.g. 120 projection 4 angular position refers to moving the arrays to 4 distinct angular positions, then acquiring 30 projections from 30 sources along each array at each angular position for 120 projections acquired in total. We chose to limit the simulations to distinct angular positions to avoid loss of generality when assuming gantry accelerations, instead stopping at defined angular positions and acquiring off the array for a short breath hold.

The source array pre treatment scans presented here were each chosen for different considerations. The 4 angular position scan could be performed with just 2 short breath holds. The 12 and 20 angular position scans could be acquired in 6 and 10 short breath holds respectively or 6 and 10 breaths if the hardware is synchronized with patient breathing as in^44^. Note that hardware synchronization as in^44^ has been practically implemented for 20 breath scans on 30 patients in the ADAPT clinical trial^45^. The 100 angular position scan was investigated as, taking flux as proxy for dose, it would have comparable radiation exposure to the conventional scan.

In the intra-treatment scan, the gantry is kept static at ${0}^{\circ }$ consistent with Fig. 1. The 60 sources in the array centred at $+{90}^{\circ }$ and the 60 sources in the array at $-{10}^{\circ }$ are activated in sequence, with all 120 projections acquired in 1 s.

### Reconstruction methods

Reconstructions were performed with the FDK algorithm, a variation of filtered back projection adapted to cone beam geometry. Note that FDK is known to need an acquisition arc over ${180}^{\circ }$ to reconstruct an entire volume.

Reconstructions were also performed using iterative reconstruction with Total Variation (TV) regularization^46^, specifically the Total Variation with Alternating Direction Method of Multipliers (TVADMM) algorithm implemented in RTK. The TVADMM algorithm attempts to find

$$x_{{TV}} = \mathop {\min }\limits_{x} \left\{ {\left\| {Ax - d_{2}^{2} + \lambda {\text{TV}}\left( x \right)} \right\|} \right\}~$$

where $x$ is a volume, $x_{TV}$ is the TVADMM estimate of ground truth $x_{GT}$, $A$ is the forward projection model, $d$ is the measured projections, $\lambda$ is the regularization parameter and

$${\text{TV}}\left( x \right) = \mathop \sum \limits_{i,j,k} \left( {\left| {x\left( {i,j,k} \right) - x\left( {i - 1,j,k} \right)} \right|^{2} + \left| {x\left( {i,j,k} \right) - x\left( {i,j - 1,k} \right)} \right|^{2} + \left| {x\left( {i,j,k} \right) - x\left( {i,j,k - 1} \right)} \right|^{2} } \right)^{\frac{1}{2}}$$

is the total variation of $x$ i.e. the sum root square of absolute differences in the image voxels along each axis. The effect is $x_{TV}$ should match the image data while having distinct regions of internally homogeneous tissue, with the balance dictated by the size of the regularization parameter $\lambda$. The TV regularization was used to account for limited data as with the source arrays.

A unique aspect of the radiotherapy workflow is the use of pre-treatment and intra-treatment imaging. It would be reasonable to make use of the pre-treatment image during reconstruction of the intra-treatment image. For example, initializing the TVADMM algorithm with the pre-treatment image. Another approach is implemented in the Prior Image Constrained Compressed Sensing (PICCS) algorithm^47,48^. The PICCS algorithm attempts to find

$$x_{{PICCS}} = \mathop {\min }\limits_{x} \left\{ {\left\| {Ax - d_{2}^{2} + \lambda \left( {\alpha {\text{TV}}\left( x \right) + \left( {1 - \alpha } \right){\text{TV}}\left( {x - x_{{pri}} } \right)} \right)} \right\|} \right\}$$

where $x_{PICCS}$ is the PICCS estimate of $x_{GT}$, $0 < \alpha < 1$ is the control parameter and $x_{pri}$ is the prior image, which we will be taking as the pre-treatment free-breathing TVADMM reconstruction.

### Image quality quantification

To quantify the per-pixel error in reconstructed images we compute the Root-Mean-Square-Error (RMSE) as $RMSE = \sqrt {\frac{1}{N}\sum \left( {x_{recon} - x_{gt} } \right)^{2} }$ where $x_{recon}$ are the reconstructed voxel values, $x_{gt}$ the true values from the XCAT phantom, and $N$ the total number of voxels in a wide region selected well contained in the CBCT field of view.

To quantify how well larger structures in the images are captured, we compute the Structural SIMilarity (SSIM)^49^ as $SSIM = \frac{1}{M}\mathop \sum \limits_{M} \frac{{\left( {2\mu_{recon} \mu_{gt} + c_{1} } \right)\left( {2\sigma_{recon,gt} + c_{2} } \right)}}{{\left( {\mu_{recon}^{2} + \mu_{gt}^{2} + c_{1} } \right)\left( {\sigma_{recon}^{2} + \sigma_{gt}^{2} + c_{2} } \right)}}$ over M windows inside the region, $\mu_{recon}$ and $\mu_{GT}$ are voxel value means in that window, $\sigma_{recon}$, $\sigma_{GT}$ and $\sigma_{recon,GT}$ are standard deviation and joint deviation, $c_{1} = \left( {0.1L} \right)^{2}$ and $c_{2} = \left( {0.3L} \right)^{2}$ are weighting factors with $L$ the dynamic range in this case $2^{16} - 1$ as reconstructions computed to 16 bit precision.

To quantify how well tissue types can be distinguished, we compute the Contrast to Noise Ratio (CNR) between the tumor and nearby homogeneous lung as $CNR= \frac{{\mu }_{lung}-{\mu }_{tumor}}{\sigma }$ where ${\mu }_{lung}$ and ${\mu }_{tumor}$ are averages of $5\times 5\times 5$ voxel regions of lung and tumor and $\sigma$ the joint standard deviation.

To quantify how well tumor edges are defined and motion accounted for, pixel profiles were taken across each axis and RMSE computed. Central slice tomographs and pixel profiles are provided in the results for qualitative evaluation. Correct identification of edges in the profiles would demonstrate a method is suitable for confirming tumour location in either pre-treatment or intra-treatment imaging i.e. within a treatment breath hold.

## Results

**Figure 2:**
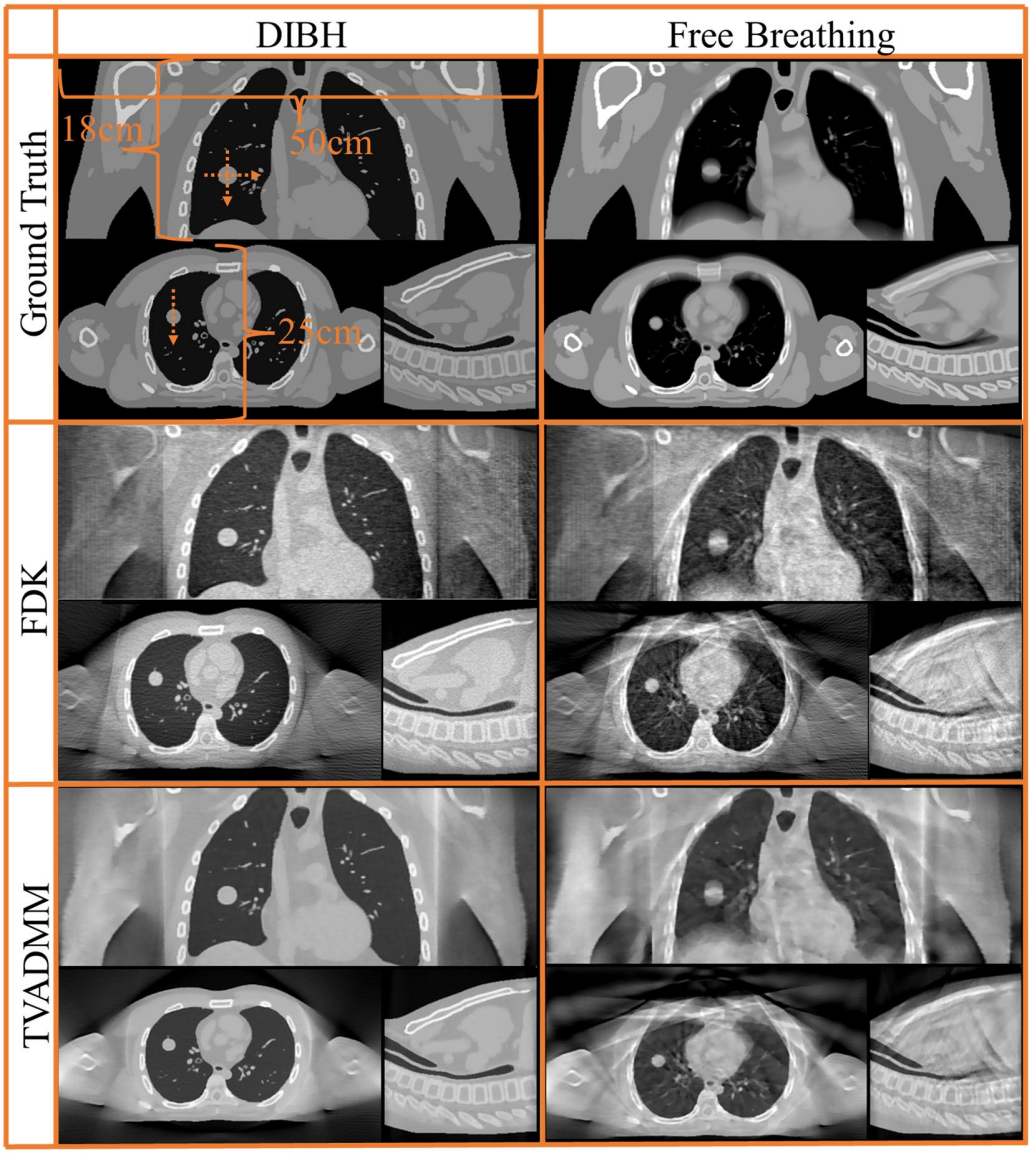
Ground truths and reconstructed pre-treatment conventional source CBCT images for Deep Inspiration Breath Hold (DIBH) and free breathing phantoms. Shown here are central slice tomographs, also defined as isocenter. Note that tumour centroid and isocenter share coronal and axial but not sagittal plane. Dashed arrow on DIBH ground truth indicate where pixel profiles were evaluated.

**Figure 3:**
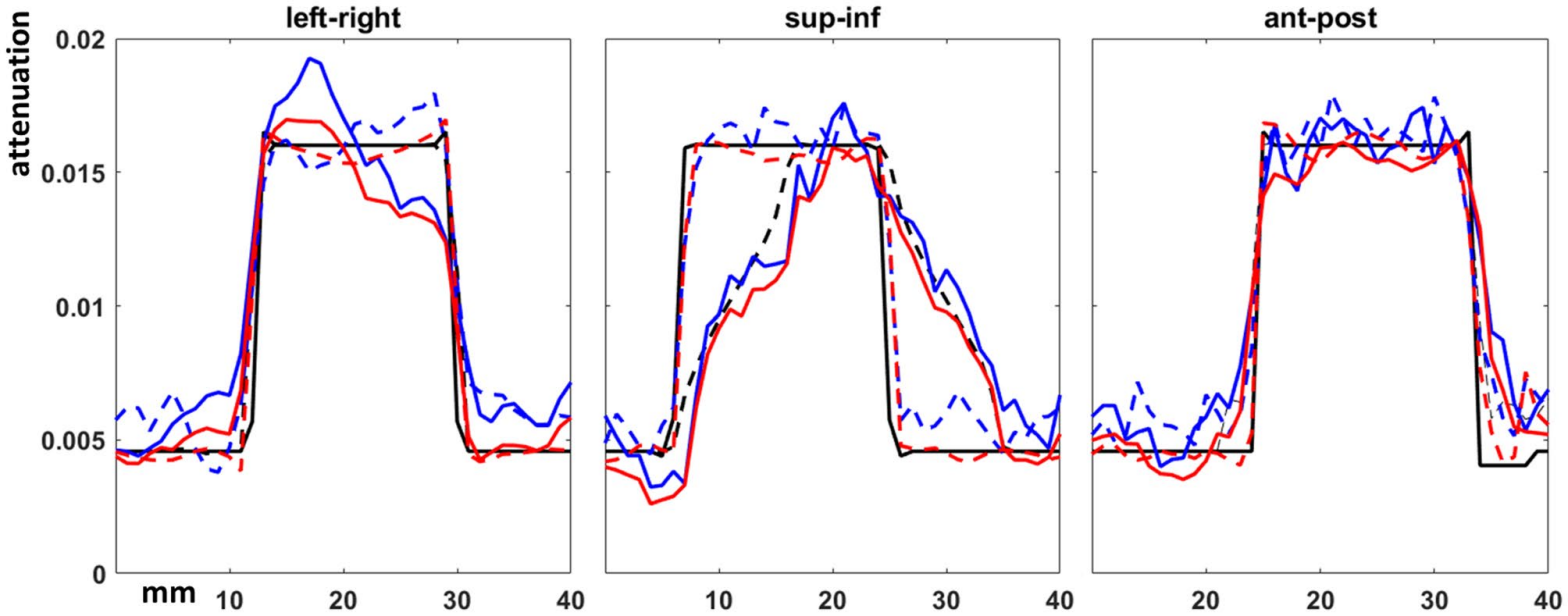
Pixel profiles across each axis of the tumor centroid of pre-treatment conventional source CBCT images. The DIBH ground truth as solid black line, free breathing ‘ground truth’ as dashed black line, DIBH FDK as solid red line, free breathing FDK as dashed red line, DIBH TVADMM as solid blue line, free breathing TVADMM as dashed blue line.

**Figure 4:**
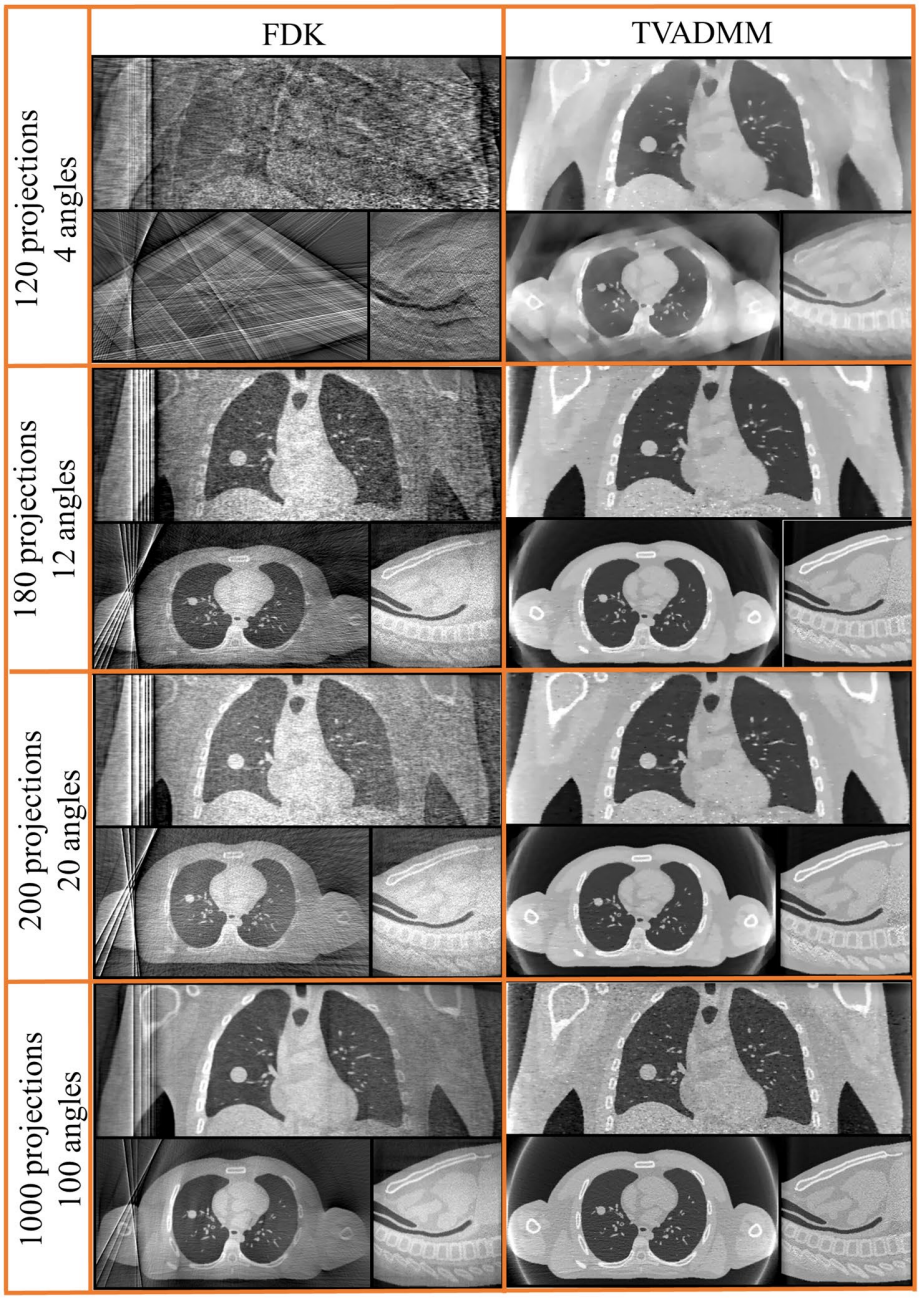
Reconstructed pre-treatment source array DIBH CBCT images for a variety of scan configurations. Note that e.g. 120 projection 4 angular position refers to moving the arrays to 4 distinct angular positions, then acquiring 30 projections from 30 sources along each array at each angular position for 120 projections acquired in total.

**Figure 5:**
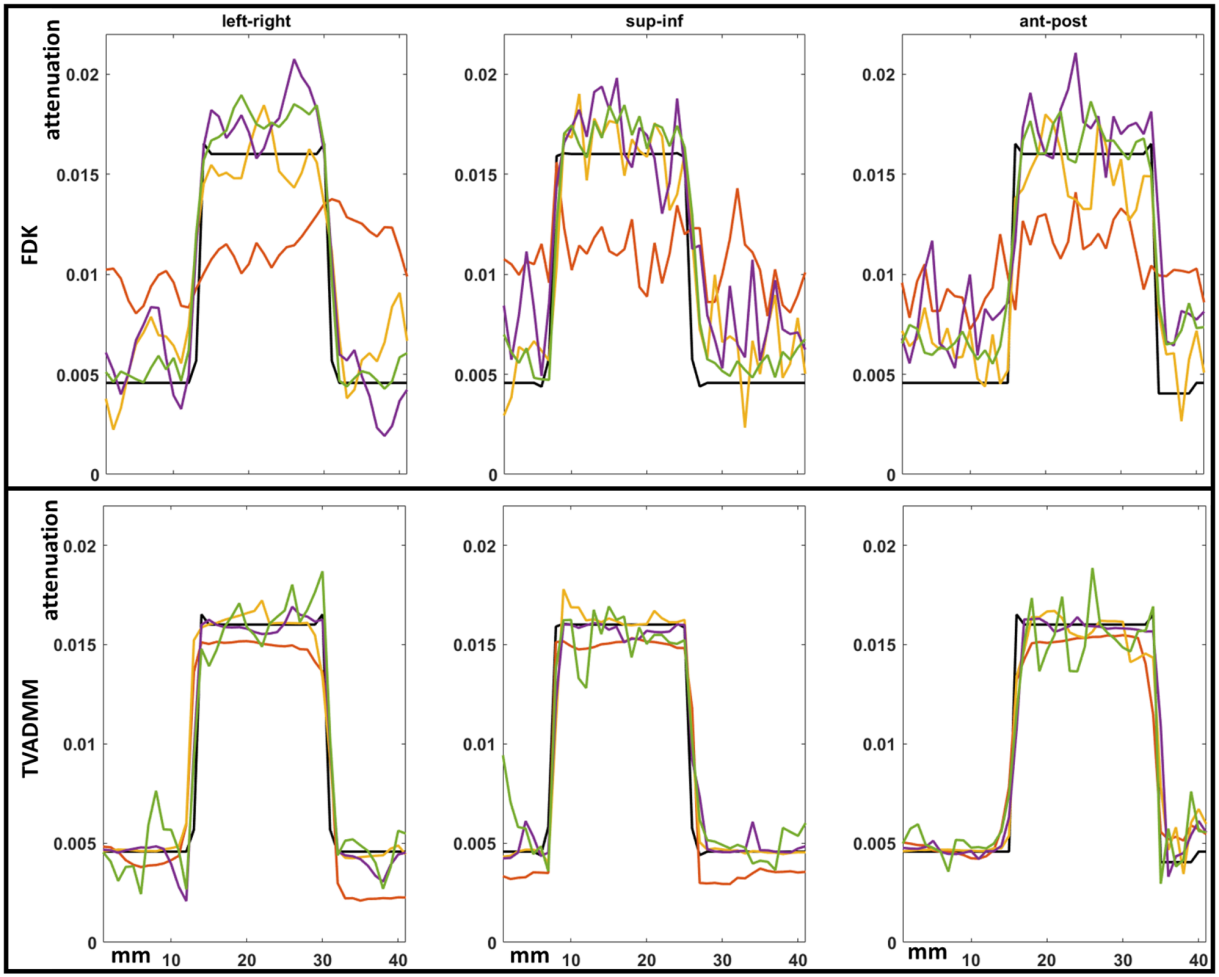
Pixel profiles across each axis of the tumor centroid of pre-treatment array source reconstructions. Top row shows FDK reconstruction results, bottom row TVADMM reconstruction results. The DIBH ground truth as black line, the 120 projection 4 angular position scan as red line, 180 projection 12 angular position scan as yellow line, 200 projection 20 angular position scan as purple line, 1000 projection 100 angular position scan as green line. Note that e.g. 120 projection 4 angular positions refers to moving the arrays to 4 distinct angular positions, then acquiring 30 projections from 30 sources along each array at each angular position for 120 projections acquired in total.

**Figure 6:**
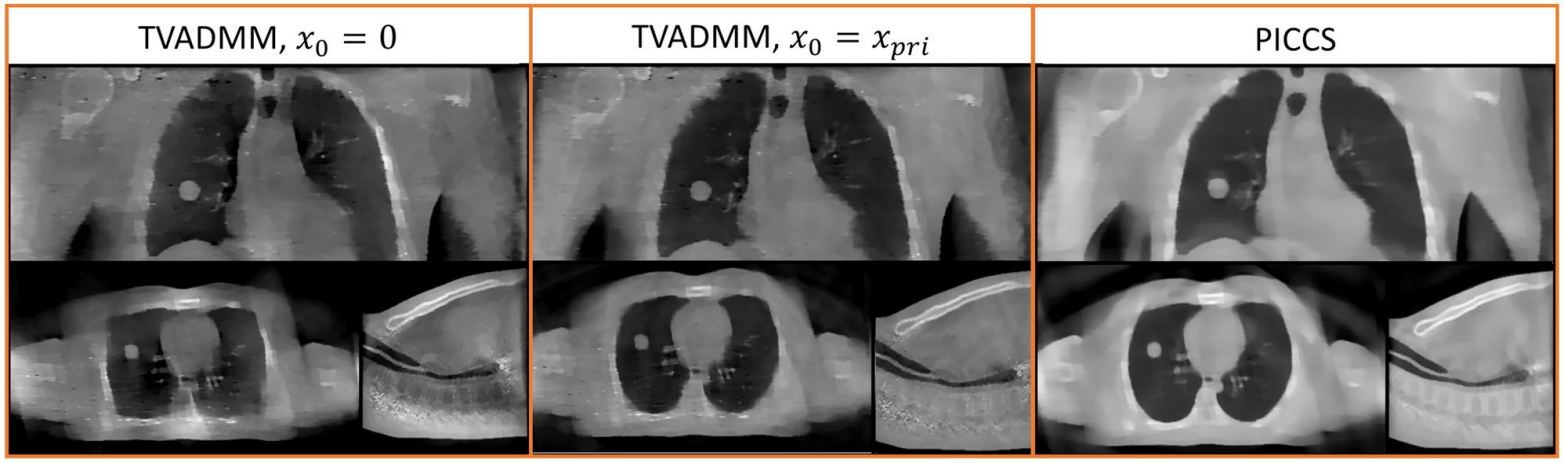
Reconstructed intra-treatment source array images. Note that ${x}_{pri}$ is the TVADMM free-breathing CBCT image.

**Figure 7:**
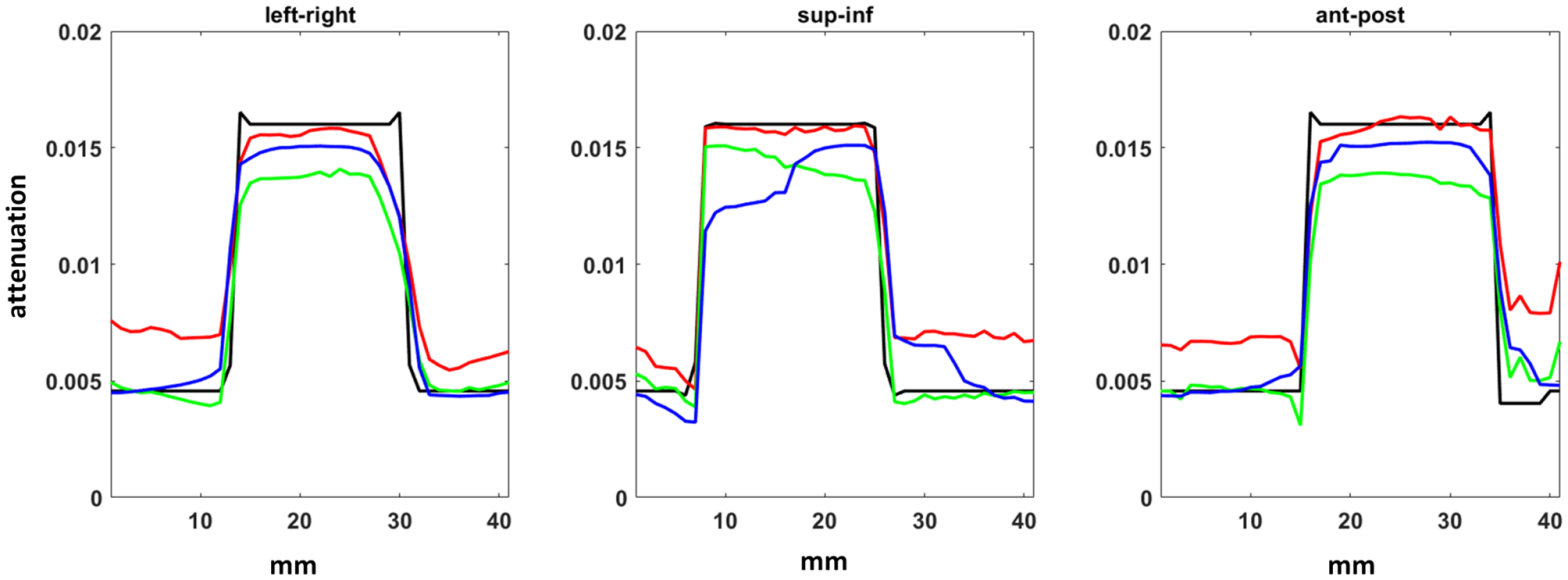
Pixel profiles across each axis of the tumor centroid from intra-treatment source array imaging. The ground truth as black line, TVADMM with ${x}_{0}=0$ as red line, TVADMM with ${x}_{0}={x}_{pri}$ as green line, PICCS as blue line.

Central slice tomographs and pixel profiles across the tumour centroid are provided in Figs. 2, 3, 4, 5, 6, 7. Results are provided in order as conventional source pre-treatment imaging, array source pre-treatment imaging, and array source intra-treatment imaging. Metrics are provided in Table 2.

**Table 2:** Quantitative metrics for each reconstruction.

	RMSE	SSIM	Tumor Profile RMSE, left–right	Tumor Profile RMSE, Sup-Inf	Tumor Profile RMSE, Ant-Post
Conventional source pre-treatment					
FDK, Free Breathing	0.00310	0.97737	0.00221	0.00480	0.00229
FDK, DIBH	0.00158	0.99488	0.00170	0.00151	0.00171
TVADMM, Free Breathing	0.00287	0.98032	0.00173	0.00474	0.00201
TVADMM, DIBH	0.00095	0.99881	0.00100	0.00127	0.00105
Array source pre-treatment					
FDK, 120 projections, 4 angles	0.00544	0.91681	0.00570	0.00542	0.00484
FDK, 180 projections, 12 angles	0.00301	0.98229	0.00224	0.00233	0.00201
FDK, 200 projections, 20 angles	0.00295	0.98295	0.00229	0.00306	0.00326
FDK, 1000 projections, 100 angles	0.00201	0.99205	0.00174	0.00183	0.00212
TVADMM, 120 projections, 4 angles	0.00212	0.98907	0.00203	0.00150	0.00136
TVADMM, 180 projections, 12 angles	0.00128	0.99798	0.00171	0.00114	0.00117
TVADMM, 200 projections, 20 angles	0.00132	0.99785	0.00112	0.00105	0.00150
TVADMM, 1000 projections					
100 angles	0.00180	0.99564	0.00149	0.00155	0.00152
Array Source Intra-Treatment					
TVADMM,${x}_{0} = 0$	0.00336	0.96888	0.00205	0.00198	0.00148
TVADMM,${x}_{0} ={x}_{pri}$	0.00227	0.98956	0.00179	0.00139	0.00223
PICCS	0.00208	0.99093	0.00243	0.00210	0.00142

### Pre-Treatment Imaging

Consider the conventional CBCT images presented in Fig. 2. Images reconstructed with TVADMM are qualitatively clearer than images reconstructed with FDK and this improvement is reflected in the image quality metrics. Note that the improvement is less marked in the free breathing scan, with motion induced artifacts apparently dominating the gains in soft tissue contrast.

Pixel profiles across the tumor centroid visible in Fig. 2 are plotted in Fig. 3. As expected, the most notable deviation from the DIBH ground truth can be seen in the Superior-Inferior pixel profile taken from the free breathing images, as this is the axis the tumor moves during free breathing acquisition. A shift is also visible in the left–right free breathing pixel profiles, possibly arising from the gantry angle/respiratory phase combinations for this patient’s breathing trace. In the DIBH scans, both TVADMM and FDK reconstructions capture the edges well.

Compare the array CBCT images presented in Fig. 4 to the single source DIBH CBCT images in Fig. 2. We note the ring around the patient, most visible in the axial slice of the TVADMM reconstruction of the 1000 projection 100 angular position scan. This is the most visible part of the truncation artefact caused by having material visible in some but not all projection images. Note that truncation artefacts effect voxel values throughout the entire reconstruction, not just the edge of the CBCT field of view. The reconstruction seems well defined within this entire region, measured to be 490 mm wide as opposed to 280 mm for the conventional source images. This increase in field of view size is one of the benefits of array CBCT. The ROI for RMSE and SSIM calculation did not include these truncation artefacts, being well contained in the CBCT field of view. The FDK reconstructions of this data seems to have similar truncation artefacts, however manifesting as vertical planes on the left of the image near the patient shoulder, effectively giving a narrower usable field of view relative to the TVADMM reconstructions. Note that these artefacts disappear for a $360^{ \circ }$ arc, however we restricted this study to a $200^{ \circ }$ arc as this is achievable on the majority of current linac gantries.

The 120 projection 4 angular positions array acquisition with TVADMM reconstruction begins to approach conventional acquisition FDK reconstruction image quality but note that the 120 projection 4 angular positions acquisition would only take two 0.5 s breath holds. This should enable fast DIBH CBCT scans for a very large patient population. The 120 projection 4 angular positions TVADMM image has 34% worse RMSE than the conventional source FDK image however in 2 of 3 axes the tumor pixel profiles are more accurate in the array-based image.

The 180 projection 12 angular positions scan was found to be the minimal number of angular positions for usable FDK reconstruction images. Note that the FDK reconstruction appears brighter near isocentre, a pattern repeated among the higher projection count FDK reconstructions. This was not observed in ${360}^{\circ }$ acquisition arcs. The relatively minor artefacts in the FDK reconstructions are surprising considering the algorithm is derived for single source circular arc acquisitions. The TVADMM reconstruction of the 180 projection 12 angular positions scan outperforms our conventional acquisition FDK reconstruction images, but not conventional acquisition TVADMM reconstruction images. This is largely due to the 80% lower photon flux in array projections, leading to greater noise in the projections and in turn the reconstructions for a given number of projections.

The 200 projection 20 angular positions scan can be seen as an array based alternative to the 20 breath respiratory motion guided 4DCBCT scan investigated in^45^. This acquisition would take 10 breaths i.e. 33 s based on^50^. Note that a respiratory motion guided 20 breaths conventional source 4DCBCT scan with perfect motion compensation would be identical to the 200 projection conventional source DIBH acquisition FDK reconstruction image shown in Fig. 3. The 200 projection 20 angular position array acquisition with FDK reconstruction slightly underperforms relative to a 200 projection conventional acquisition with FDK reconstruction in all metrics. By all metrics the 200 projection 20 location array acquisition with TVADMM reconstruction has quantitatively higher image quality than the 200 projection conventional acquisition with FDK reconstruction, but worse than the 200-projection conventional acquisition with TVADMM reconstruction image, likely due to the 80% lower photon flux.

The 1000 projection 100 angular positions array CBCT scan has equivalent photon flux to a 200 projection conventional source CBCT scan. The short arc artefacts are still present in the FDK reconstruction, and the FDK reconstruction still underperforms the gold standard conventional source DIBH acquisition FDK reconstruction image. The 1000 projection 100 angular position array acquisition with TVADMM reconstruction image also has lower quality than the conventional source DIBH acquisition TVADMM reconstruction image despite the equivalent photon flux. However, the greater FOV from array acquisition means a larger number of photons are passing through unattenuated air, so the flux through the patient is still lower, likely resulting in the lower image quality. This is one of the shortfalls of using flux as a proxy for dose.

### Intra-treatment imaging

Intra-treatment imaging results are shown in Fig. 6, replicating acquisition with the layout described in Fig. 1. Note that this acquisition assumes no motion of the gantry, just digitally switching the sources in the arrays. We exclude FDK reconstructions from the intra-treatment acquisitions as the image quality was found to be unusable.

Note that the intra-treatment imaging includes PICCS reconstructions, which include a prior image in the regularization term. We use the pre-treatment free-breathing TVADMM reconstruction image as the prior. We also include TVADMM reconstruction images initialized with the pre-treatment free-breathing TVADMM reconstruction image. The images reconstructed incorporating prior images have discernible anatomy across the entire volume, however motion blur is visible, inherited from the prior image.

Qualitatively and quantitatively, the intra-treatment imaging underperforms the conventional source pre-treatment DIBH acquisition FDK reconstruction images. Anatomy can be qualitatively resolved without incorporating prior image information, and the pixel profiles show the tumor edges are well captured. The TVADMM ${x}_{0}=0$ image is quantitatively worse than the gold standard conventional source DIBH acquisition FDK reconstruction image by all metrics except RMSE in the anterior–posterior pixel profile, and only slightly worse over the ROI than the conventional source free breathing acquisition FDK reconstruction image and with better RMSE across the tumor in all axes. The TVADMM ${x}_{0}={x}_{pri}$ and PICCS images are quantitatively better than the conventional source free breathing acquisition FDK reconstruction image, but still slightly worse than the gold standard conventional source DIBH acquisition FDK reconstruction image except for along some pixel profile axes.

Intra-treatment pixel profiles are provided in Fig. 7. Note that the gantry is at ${0}^{\circ }$ as in Fig. 1 so intra-treatment imaging would be for guiding the beam is specifically the left–right and superior-inferior axes. The anterior–posterior pixel profile is also provided for completeness but is not strictly relevant to beam guidance at this gantry angle.

## Discussion

This study investigated several novel source array configurations for imaging in thoracic radiotherapy. These novel source arrays have the potential to reduce scan times and improve image quality in the radiotherapy context. However, we note that image quality requirements are application specific. Conventional source free breathing acquisition FDK reconstruction images are acceptable for certain patients depending on treatment fractionation, tumour location and motion, but improved motion management would expand the eligible patient cohort for emerging high dose per treatment and high dose rate methods^3,7,8^.

Although the TVADMM and PICCS reconstruction image quality is satisfactory for several clinical applications, the computation time is relatively high, taking several minutes in our implementation as opposed to near real time for FDK when memory overheads are accounted for. In this study we investigated these compressed sensing type algorithms as a proxy for investigating whether a proposed acquisition contains “sufficient” data for adequate image quality. Clinical implementation would require computational improvements, for example replacing such algorithms with a neural network has been successful in the context of real time MRI^28,51–54^ and will be investigated in future. We present figures with no post processing to best represent the underlying differences in each acquisition protocol and reconstruction algorithm while noting that these artefacts could be easily removed for clinical application.

Note that we limited our investigation to full fan acquisition with a gantry only capable of ${200}^{\circ }$ rotation. This is the simplest configuration, however practical integration of source arrays should consider the full gantry capabilities e.g. wider field of view if the detector can be offset and the gantry can rotate ${360}^{\circ }$.

The proposed hardware consists of two 60 cm long source arrays and two detectors. We analysed using combinations of a conventional source, one source array, and one or two detectors, but found anything less than the configuration proposed in Fig. 1 did not acquire sufficient information for intra-treatment imaging. Information regarding these partial approaches has been removed for the sake of focussing the manuscript.

### Pre-treatment imaging

Pre-treatment source array imaging is shown in Fig. 4. Based on the image quality metrics, 120 projection 4 angular position source array acquisition TVADMM reconstruction imaging would be acceptable for pre-treatment imaging as it outperforms conventional source free breathing acquisition FDK reconstruction imaging. This data could be acquired in 2 breaths in using breath holds or adaptive acquisition as in^45^. Qualitatively however artefacts are clearly present in the reconstructed image.

One of the arguments for continued use of FDK in clinical imaging is that TVADMM reconstruction can smooth out real anatomical features^55^. For this reason, we increased the pre-treatment imaging angular positions until a meaningful FDK image was obtained. Quantitatively the 180 projection 12 angular position source array acquisition with FDK reconstruction has improved image quality relative to the conventional source free breathing acquisition with FDK reconstruction. However, even the 1000 projection 100 angular position source array acquisition with FDK reconstruction and with TVADMM reconstruction underperform the gold standard conventional source DIBH acquisition with FDK and TVADMM reconstruction images. This suggest that the reduced flux in source array imaging makes it difficult to achieve image quality at the level of conventional source imaging for patients capable of performing long breath holds.

Based on these results, the ability to perform source array acquisitions quickly relative to patient respiration thereby isolating patient motion is more important to image quality than the ability to image with high photon flux. This assumes imaging can be coordinated with patient respiration either via breath holds as in^11,14^ or prospective monitoring as in^45^.

The pre-treatment scenario presented in this study has been limited to 3D imaging. However, 4D imaging is also used widely in radiotherapy^56^. Source array imaging could be integrated with prospective 4D acquisition as in^45^ to potentially perform 4D acquisition in just 2 patient breaths as opposed to 20 breaths with a conventional source in^45^ and 240 s in standard clinical practice^43^. The need for 4D pre-treatment imaging would be reduced in source array equipped systems by the availability of intra-treatment imaging.

### Intra-treatment imaging

The reconstructed intra-treatment images are provided in Fig. 6 and pixel profiles across the tumor are provided in Fig. 7. The first note to make is that intra-treatment imaging on current standard systems varies between clinics and may include any combination of in room video cameras^14^, IR markers on the patient^23^, surface tracking of the patient^11,12,14^, low dose X-ray video using the kV imager (fluoroscopy)^14,15,34^, or MV imaging using the treatment beam and EPID^16,17^. A recent development is MR-linacs^24,25,29^ however these systems are expensive and currently restricted to only imaging a few 2D planes during treatment rather than a complete volumetric image, although volumetric imaging during treatment has been achieved in simulation and research environments^26,28,54^.

The intra-treatment PICCS and the less restrictive TVADMM with ${x}_{0}={x}_{pri}$ image appear qualitatively good. However, the pixel profiles reveal that inheriting information from the prior brings motion blur which may complicate beam guidance. This issue may be reduced by using a respiratory correlated 4D image as a prior, but this requires a 4D pre-treatment scan and assumes a level of agreement between pre-treatment and intra-treatment anatomy.

Recall the hardware layout from Fig. 1. When treating at this angle, the left–right and superior-inferior accuracy is most important. Interestingly the tumor profile shows that the proposed configuration captures the tumor location well in the left–right and superior-inferior axes even without a prior image. This is because the treatment delivery plane and tomosynthesis plane in this configuration are only offset ${10}^{\circ }$. We believe this is a promising result for intra-treatment beam guidance.

The intra-treatment imaging qualitatively matches the entire volume without referring to a prior image. The image is qualitatively and quantitatively worse than typical pre-treatment images. However, the proposed configuration can acquire in 1 s without moving the gantry. So although image quality is worse, we believe intra-treatment image quality would be sufficient for intra-treatment guidance given the tumor definition as seen from the pixel profiles. Surrounding tissue may also be reconstructed to sufficient accuracy for retrospective dosimetry, e.g., to check that nearby organs at risk were not receiving unacceptably high radiation doses during the treatment delivery. While we believe prior image guided intra-treatment imaging would work well for the vast majority of cases, knowing that our proposed configuration can volumetrically image with much weaker assumptions on the underlying image may be worth the extra hardware and exposure cost.

## Conclusion

We have proposed a novel hardware configuration integrating source arrays into conventional radiotherapy linacs. We have investigated imaging capabilities and showed that this configuration is feasible and offers benefits over the current generation of radiation therapy machine at both the pre-treatment and intra-treatment stage. Our proposed hardware would reduce pre-treatment scan times and expand the patients eligible for breath hold imaging, halving the required gantry rotation and able to acquire with breath holds of just 1 s or synchronized with 2–10 free breathing cycles as in^43–45^. Integrating two source arrays and two high frame rate detectors with an otherwise typical linac gantry was found to enable volumetric imaging during treatment delivery, which may reduce uncertainty in beam guidance and retrospective dosimetry to acceptable levels for emerging high dose rate^5,7^ or dose per treatment^1^ radiotherapy for a wide patient cohort^8,56^. These results motivate construction of a physical prototype and further developments in source array imaging hardware and software.

## Data Availability

All codes and parameter files used for this study are available on reasonable request to the corresponding author.
